# Case Report: Primary small bowel adenocarcinoma with peritoneal metastasis responded well to a CapeOX + bevacizumab regimen

**DOI:** 10.3389/fgstr.2023.1187194

**Published:** 2023-08-21

**Authors:** Guang Fu, Zhen Tang, Zishun Xu, Shao Zhang

**Affiliations:** The First Affiliated Hospital, Department of Gastrointestinal Surgery Hengyang Medical School, University of South China, Hengyang, China

**Keywords:** small bowel adenocarcinoma, peritoneal metastasis, perforation, CapeOX, bevacizumab

## Abstract

**Background:**

Small bowel adenocarcinoma (SBA) is a rare condition often presenting with various non-specific gastrointestinal symptoms, making its diagnosis challenging. Delayed diagnosis is common, as patients may not receive the correct diagnosis until complications arise, necessitating further investigations. Furthermore, the management of SBA patients poses difficulties due to the scarcity of high-quality evidence.

**Case presentation:**

In this report, we present the case of an elderly man with SBA in the ileum who arrived at our emergency room with acute abdominal pain. The diagnosis was not made until the SBA caused a perforation, leading to acute abdominal pain. An emergent exploratory laparotomy revealed a 3 cm × 3 cm perforated tumor in the ileum, along with widespread metastatic nodules on the omentum, ascending colon, descending colon, and rectum. Postoperative pathological evaluation confirmed the diagnosis of SBA with peritoneal metastasis (pT4N2M1, stage IV). Following surgery, the patient received palliative systemic chemotherapy, which included the CapeOX regimen and the anti-VEGF monoclonal antibody bevacizumab. Remarkably, the patient responded well to this therapy, displaying good tolerance, and we observed no signs of disease progression. As of now, the patient is in good health and continuing with regular follow-up.

**Conclusion:**

The early diagnosis of small bowel adenocarcinoma remains a challenge. Delayed diagnosis can lead to a poor prognosis, underscoring the importance of considering SBA as a potential diagnosis for patients with unexplained abdominal pain and gastrointestinal symptoms. This case also highlights the efficacy of palliative chemotherapy with the CapeOX regimen combined with bevacizumab in controlling SBA.

## Introduction

Although the small intestine accounts for three-quarters of the entire length of the intestinal tract and plays a crucial role in absorption, malignancies affecting this organ account for less than 5% of all gastrointestinal cancers (1). The annual incidence of small bowel malignancies is estimated to be less than two cases per 100,000 individuals (2). Among these, small bowel adenocarcinoma (SBA) is the most prevalent histologic type, comprising approximately 30%–40% of all small bowel cancers. Carcinoid tumors account for 35%–42%, lymphoma for 15%–20%, and sarcoma for 10%–15% (3–6). Notably, SBA is predominantly found in the duodenum (60%), with involvement of the jejunum (30%) and ileum (10%) being less common (5, 7–10). Consequently, encountering SBA in the ileum is rare in clinical practice.

Patients with SBA present with various non-specific gastrointestinal symptoms, including chronic abdominal pain or discomfort, nausea, vomiting, gastrointestinal bleeding, bowel obstruction, and also non-specific systemic symptoms such as anemia and weight loss (1, 8). Currently, there are no specific presentations exclusively attributed to SBA patients. As a result, many SBA patients are misdiagnosed and receive irrelevant medical care. In advanced stages, SBA can lead to complications such as jaundice, bowel obstruction, and perforation, the severity of which depends on the tumor’s location, size, and stage (7, 11). Unfortunately, some SBA patients do not receive a correct diagnosis until these complications necessitate further diagnostic investigations. Moreover, conventional radiological imaging studies have limited sensitivity in detecting small bowel tumors (12, 13). These factors contribute significantly to delayed diagnosis of SBA, sometimes up to 2 years after the initial onset of symptoms (14). Therefore, diagnosing SBA in its early stages poses challenges and as it may be elusive during the initial encounter. Consequently, many SBA patients present to the emergency department with an advanced tumor stage complicated by bowel obstruction and perforation (11). Unfortunately, patients with delayed diagnoses have missed the opportunity for early intervention and treatment, resulting in a very poor prognosis with a limited 5-year survival rate of 14%–33% (15, 16).

In this case report, we describe an elderly man with SBA in the ileum who presented to our emergency department with acute abdominal pain. His SBA diagnosis was delayed until the onset of acute abdominal pain caused by tumor perforation. Previously, he had been misdiagnosed with irritable bowel syndrome and prescribed medication. His final diagnosis was made more than 4 months after the initial onset of abdominal pain.

## Case presentation

**Figure 1:**
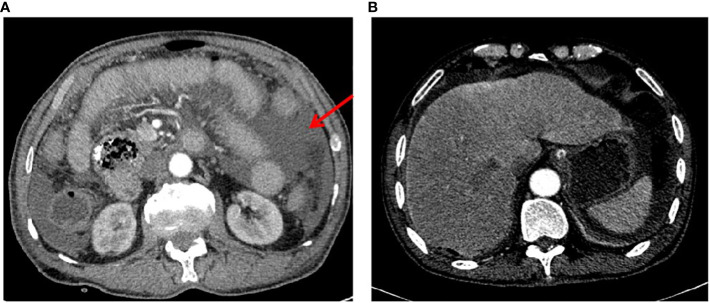
Preoperative enhanced CT scan images. **(A)** A typical image highlighting the presence of effusion in the abdominal cavity, as marked by the red arrow. **(B)** A representative image demonstrating the absence of metastatic lesions in the liver.

A 73-year-old male patient presented to our emergency room with acute and diffuse abdominal pain accompanied by a high fever (39°C). He denied experiencing vomiting, nausea, diarrhea, constipation, or radiating pain, but physical examination revealed diffuse tenderness, a rigid abdomen, rebound tenderness, and shifting dullness. Four months earlier, he had experienced periumbilical abdominal pain and underwent a gastroduodenoscopic examination, which led to a diagnosis of chronic non-atrophic gastritis and duodenal diverticula. He was prescribed proton pump inhibitors (PPI) and probiotics, but his symptoms showed only slight improvement and worsened in the 4 days preceding admission. After being admitted to a local hospital and undergoing an abdominal X-ray, which showed no abnormalities, he was transferred to our hospital’s emergency room due to sudden abdominal pain and a high fever 1 day prior. The patient’s medical records and physical examination raised suspicion of gastrointestinal tract perforation. An enhanced CT scan was performed, revealing significant accumulation of fluid and air within the abdominal cavity, confirming the clinical suspicion of gastrointestinal perforation (**Figure 1A**). No liver nodules were detected on the enhanced CT scan (**Figure 1B**). However, mild dilation of the jejunum was observed without any other anomalies. Laboratory tests revealed anemia and hypoproteinemia, and tumor-associated antigen screening showed elevated levels of CA19-9 and CA12-5.

**Figure 2:**
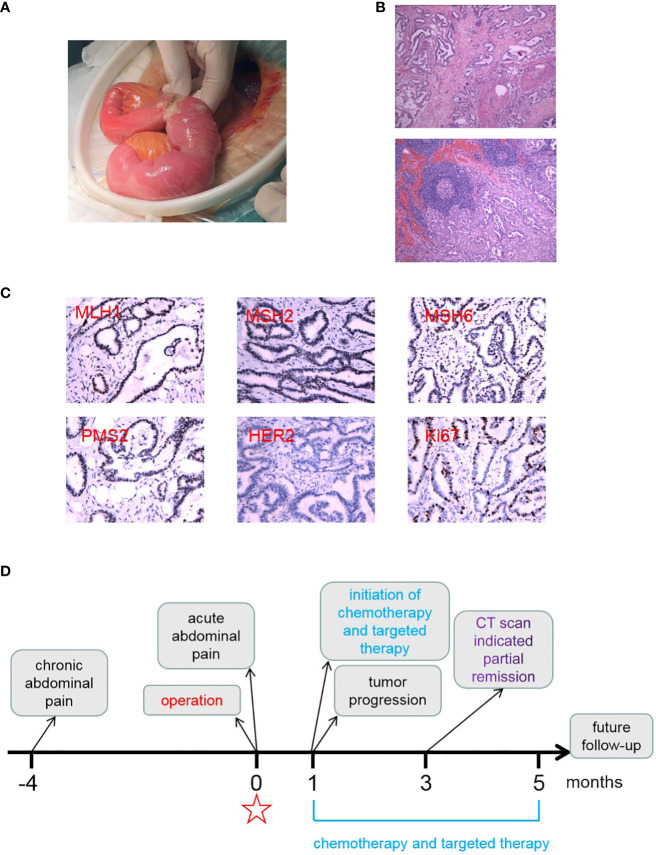
Postoperative pathologic examination of the patient’s tumor specimen and an illustrative timeline of our treatment and patient’s response. **(A)** A photo displaying the perforation of the tumor in the ileum during the operation. **(B)** Representative photos of the tumor specimen with hematoxylin and eosin staining, revealing moderately to poorly differentiated adenocarcinoma. **(C)** A representative photo of immunohistochemistry staining indicating positive expression for MLH1, MHS2, MSH6, PMS2, and Ki67, but negative expression for Her2. **(D)** An illustrative timeline showcasing our treatment approach and the patient’s response.

Due to the suspected gastrointestinal tract perforation and severe peritonitis, emergent exploratory laparotomy was recommended and performed. During the examination of the small intestine, a perforated tumor measuring 3 cm × 3 cm in size, with indistinct margins, was identified in the ileum (**Figure 2A**). The tumor had invaded the serosa and surrounding mesentery. A comprehensive intraoperative inspection of the peritoneal cavity and visceral organs revealed the diffuse presence of suspected seeding metastatic nodules on the omentum, ascending colon, descending colon, and rectum. A liver nodule, which was highly suspected to be metastatic, was identified and excised for pathological examination. Surprisingly, the pathological examination revealed that the liver nodule was a hepatic hemangioma and not a metastasis. The tumor, regional lymph nodes, and involved mesentery were resected during the operation. Subsequent postoperative pathological examination confirmed that a moderately to poorly differentiated adenocarcinoma (**Figure 2B**) had invaded the muscular layer and reached the extraserous fat. Tumor emboli were observed in the vessels, but no nerve invasion was detected. The surgical margins were clear of adenocarcinoma residue, although metastatic nodules were found in the mesentery and omentum, and metastasis was observed in 5 out of 18 mesenteric lymph nodes. The adenocarcinoma was staged as pT4N2M1 (stage IV).

Immunohistochemistry staining of mismatch repair-related proteins yielded the following results: MLH1 (mutL homolog 1) positive (+), MSH2 (mutS homolog 2) positive (+), MSH6 (mutS homolog 6) partially positive (+), and PMS2 (PMS1 homolog 2) positive (+) (**Figure 2C**). The tumor tested negative for Her2 (human epidermal growth factor receptor 2) and showed approximately 30% positivity for Ki67 (**Figure 2C**). Gene mutation screening revealed that the patient had microsatellite stability and no mutations in *BRAF* (vrafmurine sarcoma viral oncogene homolog B), *ERBB2* (erb-b2 receptor tyrosine kinase 2), *KRAS* (Kirsten rat sarcoma viral oncogene homolog), *NRAS* (neuroblastoma RAS viral oncogene homolog), *NTRK1* (neurotrophic receptor tyrosine kinase 1), *NTRK2* (neurotrophic receptor tyrosine kinase 2), and *NTRK3* (neurotrophic receptor tyrosine kinase 3) (**Table 1**).

**Table 1:** Results of gene mutation screening by next-generation sequencing.

Gene	*BRAF*	*ERBB2*	*KRAS*	*NRAS*	*NTRK1*	*NTRK2*	*NTRK3*	*EGFR*
**Wild type**	√	√	√	√	√	√	√	√
**Mutation**								

No mutations were detected in the BRAF, ERBB2, KRAS, NRAS, NTRK1, NTRK2, NTRK3, and EGFR genes.

BRAF, vrafmurine sarcoma viral oncogene Homolog B; KRAS, Kirsten rat sarcoma viral oncogene homolog; NRAS, neuroblastoma RAS viral oncogene homolog; ERBB2, erb-b2 receptor tyrosine kinase 2; NTRK1, neurotrophic receptor tyrosine kinase 1; NTRK2, neurotrophic receptor tyrosine kinase 2; NTRK3, neurotrophic receptor tyrosine kinase 3; EGFR, epidermal growth factor receptor.

**Figure 3:**
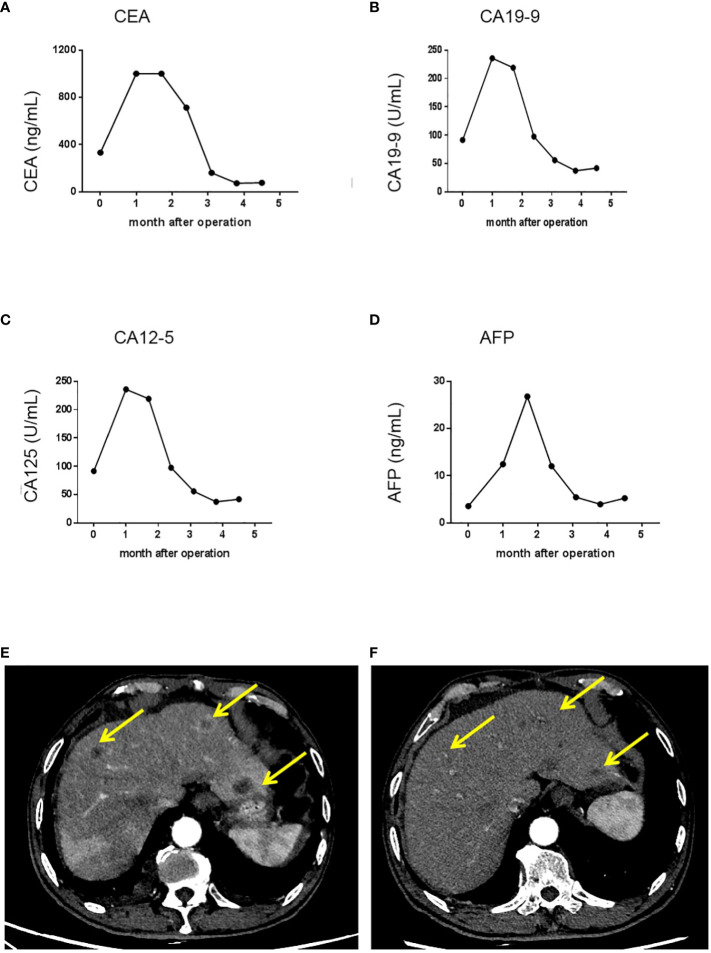
Trends in serum tumor marker levels and shrinkage of liver metastatic nodules after the initiation of chemotherapy and targeted therapy. **(A)** Serum CEA levels in each cycle of therapy; **(B)** Serum CA19-9 levels in each cycle of therapy; **(C)** Serum CA12-5 levels in each cycle of therapy; **(D)** Serum AFP levels in each cycle of therapy. **(E)** A representative image showing liver metastatic nodules found one month after the operation. **(F)** A representative image showing shrinkage of liver metastatic nodules. After initiation of chemotherapy and targeted therapy. CA 19-9, carbohydrate antigen 19-9; CEA, carcino-embryonic antigen; CA12-5, carbohydrate antigen 12-5; AFP, alpha fetoprotein.

Based on the tumor stage, postoperative systemic chemotherapy was strongly recommended. In addition, iron supplements were administered to address the patient’s anemia before commencing chemotherapy. The patient had a smooth recovery following the operation. However, 1 month later, he experienced rapid disease progression, as evidenced by a significant increase in serum CEA, CA19-9, CA12-5, and AFP levels (**Figures 2D**, **3A–D**). An enhanced CT scan revealed the presence of liver metastatic nodules (**Figure 3E**). Consequently, immediate initiation of systemic chemotherapy using the CapeOX regimen, combined with the anti-VEGF monoclonal antibody bevacizumab, was recommended.

The patient exhibited a favorable response to the therapy, as evidenced by a gradual decrease in the levels of CEA, CA19-9, CA12-5, and AFP that were observed in their serum after the initial cycle of chemotherapy and targeted therapy (**Figures 3A–D**). Follow-up CT scan results demonstrated significant shrinkage of the metastatic nodules, indicating partial remission 3 months post surgery (**Figure 3F**). Currently, the patient is in good health and experiencing only minor side effects. It is important to note that although the patient refused further chemotherapy and targeted therapy due to minor side effects, regular follow-up is still being conducted. In addition, the patient expressed great appreciation for our treatment, which alleviated his abdominal pain, stating, “Although postoperative chemotherapy and targeted therapy can be bothersome, I have no other choice but to pursue them in order to prolong and improve my survival”.

## Discussion

We present a rare case of small bowel adenocarcinoma (SBA) located in the ileum, which initially presented as acute abdomen due to perforation caused by the tumor. Regrettably, the diagnosis was significantly delayed, occurring 4 months after the patient experienced the first episode of periumbilical abdominal pain, leading to a misdiagnosis of irritable bowel disease. Currently, endoscopic techniques such as wireless capsule endoscopy or double-balloon endoscopy are essential for the evaluation of small intestine diseases beyond the proximal duodenum or terminal ileum (17, 18). Conventional CT scans have shown limited reliability in detecting SBA (19). Furthermore, the absence of specific serologic biomarkers for SBA adds to the diagnostic challenge, placing patients at a higher risk of disease progression and poorer prognosis (1, 14).

Management strategies for SBA were previously based on limited data and adopted similar approaches to those used for colorectal cancer (CRC) before the development of guidelines (20). In 2018, the French intergroup published their guidelines for the diagnosis, treatment, and follow-up of SBA (21). Subsequently, in 2019, the National Comprehensive Cancer Network (NCCN) Clinical Practice Guidelines in Oncology also released their guidelines for SBA (22). Both guidelines recommend surgical resection as the only curative strategy for localized disease, with segmental resection being the standard surgical procedure. Adjuvant chemotherapy should be used cautiously, as studies have shown conflicting results in terms of its efficacy in improving patient outcomes (23). The first-line regimen for advanced disease, as in our case, is a fluoropyrimidine-based regimen (CapeOX regimen) (24), with the addition of anti-VEGF monoclonal antibody bevacizumab (25) or anti-EGFR monoclonal antibody cetuximab (26), showing promising results. However, the immune checkpoint inhibitor pembrolizumab has demonstrated a low overall response rate (27). Based on the aforementioned data, in this case, we initiated systemic chemotherapy using the CapeOX regimen and bevacizumab after disease progression, resulting in apparent disease remission.

In our patient, we observed massive peritoneal metastases, which are more commonly encountered in SBA originating from the jejunum and ileum (28, 29). Patients with such metastases usually experience rapid disease progression and have a poor prognosis, with an estimated median overall survival of 5.9 months (28, 29). Cytoreductive surgery (CRS) and hyperthermic intraperitoneal chemotherapy (HIPEC) are optional treatments in addition to systemic chemotherapy. Several retrospective studies have primarily demonstrated their effectiveness in improving disease-free survival and overall survival (30–32). However, these studies are often limited by the number of patients and heterogeneity among them. Therefore, high-quality data from prospective studies are urgently needed to guide clinical practice.

SBA exhibits a distinct genomic profile compared with CRC, with different landscapes of genetic alterations that can influence outcomes and prognosis (33–35). SBA shows a lower incidence of adenomatosis polyposis coli (APC) alteration but a higher incidence of *Her-2* and *CDKN2A* gene alterations than CRC (33, 34). In addition, microsatellite instability (MSI) appears to be more frequent in SBA than in CRC (36), with up to 70% of celiac-associated cases exhibiting this phenotype (7). In this case, next-generation sequencing and immunohistochemistry staining did not reveal any relevant gene alterations, indicating a microsatellite stable and mismatch repair-proficient state. Consequently, this patient has a minimal likelihood of benefiting from immune checkpoint inhibitors.

## Conclusion

This case report emphasizes the difficulties involved in diagnosing and managing SBA, underscoring the necessity for additional studies to provide guidance in clinical practice. Delayed diagnosis can result in a poorer prognosis, highlighting the significance of considering SBA as a potential diagnosis in patients presenting with unexplained abdominal pain and gastrointestinal symptoms.

## Data availability statement

The original contributions presented in the study are included in the article/supplementary material. Further inquiries can be directed to the corresponding authors.

## Ethics statement

The studies involving human participants were reviewed and approved by The First Affiliated Hospital, University of South China. The patients/participants provided their written informed consent to participate in this study. Written informed consent was obtained from the participant/patient(s) for the publication of this case report.

## Author contributions

GF and SZ designed the study. ZT and ZX collected the data. GF analyzed the data and drafted the manuscript. GF and SZ performed the operation. All authors contributed to the article and approved the submitted version.
